# Krüppel-like factor 2 suppresses human gastric tumorigenesis through inhibiting PTEN/AKT signaling

**DOI:** 10.18632/oncotarget.22229

**Published:** 2017-11-01

**Authors:** Chunmei Wang, Liang Li, Qiuhui Duan, Qingqing Wang, Jinlian Chen

**Affiliations:** ^1^ East China Normal University and Shanghai Fengxian District Central Hospital Joint Center for Translational Medicine, Shanghai 200241, China; ^2^ Department of Gastroenterology, Affiliated Fengxian Hospital of Southern Medical University, Shanghai 201499, China; ^3^ Shanghai East Hospital, Tongji University School of Medicine, Shanghai 200120, China; ^4^ Department of Gastroenterology, Affiliated Fengxian Hospital of Anhui University of Science and Technology, Shanghai 201499, China

**Keywords:** KLF2, gastric cancer, prognosis, tumor suppressor, PTEN-AKT-mTOR signaling

## Abstract

Krüppel-like factors (KLFs) are a large family of DNA-binding transcriptional regulators that affect basic cellular processes such as growth, survival, migration and differentiation and serve a complicated function in cancers. KLF2, one member of the KLF family, is dysregulated in many tumors. However, the specific role of KLF2 in human gastric tumorigenesis is unknown. Here we show that the expression of KLF2 protein was lower in gastric tumors when compared with adjacent normal tissue. Moreover, downregulated KLF2 expression in primary gastric tumor was closely correlated with patients’ survival. Various cell experiments showed that ectopic KLF2 expression suppressed the proliferation, migration and invasion of gastric cancer cells. Moreover, KLF2 overexpression remarkably enhanced cell apoptosis and induced cell cycle arrest. Impaired expression of KLF2 markedly promoted cell growth *in vitro* and significantly expanded tumor size *in vivo*. Mechanically, the mRNA and protein level of PTEN was reduced in KLF2 deficient cells and xenograft tumors, suggesting that PTEN/AKT signaling was involved in the gastric tumor inhibitory effect of KLF2. Administration of AKT inhibitor AZD5363 or Insulin-like growth factor-1 (IGF-1) in KLF2 knockdown or ectopic expression cell lines, respectively, substantially reversed the proliferation phenotype. Collectively, our findings provide clinical evidence and a potential mechanism supporting that KLF2 suppresses human gastric tumorigenesis through inhibiting the PTEN/AKT axis.

## INTRODUCTION

As the fifth most common cancer worldwide, gastric cancer (GC) is a serious public health problem. There are more than 950,000 new cases diagnosed every year. In 2012, it is estimated that there were 720,000 patients died from this disease globally [1]. GC is a leading cause of cancer death worldwide because it is often diagnosed at a late stage. Though the incidence has declined in North America and major countries in western European, it remains high in Asia, especially in China [2]. Currently, the most regular and effective therapeutic choice for GC is surgical resection [3]. The poor overall survival and deficiency of effective therapy regimen for the recurrent and advanced gastric cancer patients make it important to identify novel treatment modalities for gastric cancer [4]. Thus, exploring the underlying regulators and mechanism during the GC development and progression will be valuable to develop novel targeted treatment strategy.

Emerging as an important family, KLFs are consist of a triple zinc-finger DNA-binding domain at the C-terminus and a more variable N-terminus that endows KLFs with functional diversity depending on the binding partners’ attributes. These 17 KLFs function as transcription regulators that control important cellular activities including cell differentiation, proliferation, migration and pluripotency [5-7]. In addition, it has been reported that KLFs are often dysregulated and act as tumor suppressors or oncogenes in human cancers depending on the specific tissue context, tumor type or stage [8]. KLF2, as one prominent member, is lethal when deleted in mice and is aberrantly expressed in various human tumors, such as ovarian [9], prostate [10], pancreatic [11] and non-small-cell lung cancer (NSCLC) [12]. Forced overexpression of KLF2 restrains cell growth, induces cell cycle arrest and enhances cell apoptosis [9, 10]. According to a recent report, decreased expression of KLF2 in human cancer was attributed to epigenetic silencing by the histone methyltransferase EZH2 [13]. These studies consider KLF2 as a tumor suppressor given all these characteristics. In contrast to the above results, our previous study on hepatocellular carcinoma (HCC) revealed unexpectedly that KLF2 actually works as an oncogene [14]. KLF2 promotes HCC cell proliferation by upregulation of c-myc. All these findings suggest that KLF2 has complicated but also important roles in tumorigenesis, with the underlying mechanism remaining unclear. Moreover, it is still obscure whether and, if so, how KLF2 is involved in the tumorigenesis of gastric cancer.

In this study, we investigated the expression pattern and relevant clinical features of KLF2 in human gastric cancer and the effect of its alteration on gastric cancer cell biology. We further explored the potential mechanism of KLF2 by taking advantage of the stable KLF2 human gastric cancer cell lines. The results showed that KLF2 was downregulated in human gastric cancer and directly correlated with patient survival rate. Consistent with this, impaired expression of KLF2 significantly promoted GC growth *in vitro* and tumor formation *in vivo*. KLF2 also inhibited cell migration and invasion, and induced cell cycle arrest and cell apoptosis. Mechanistically, KLF2’s anti-proliferation effect was mediated through the inhibition of PTEN/AKT signaling. Thus, our study defines KLF2 as a tumor suppressor in human gastric cancer that regulates PTEN expression to repress downstream AKT-mTOR signaling.

## RESULTS

### KLF2 is downregulated in human gastric cancer and predicts poor prognosis

**Figure 1:**
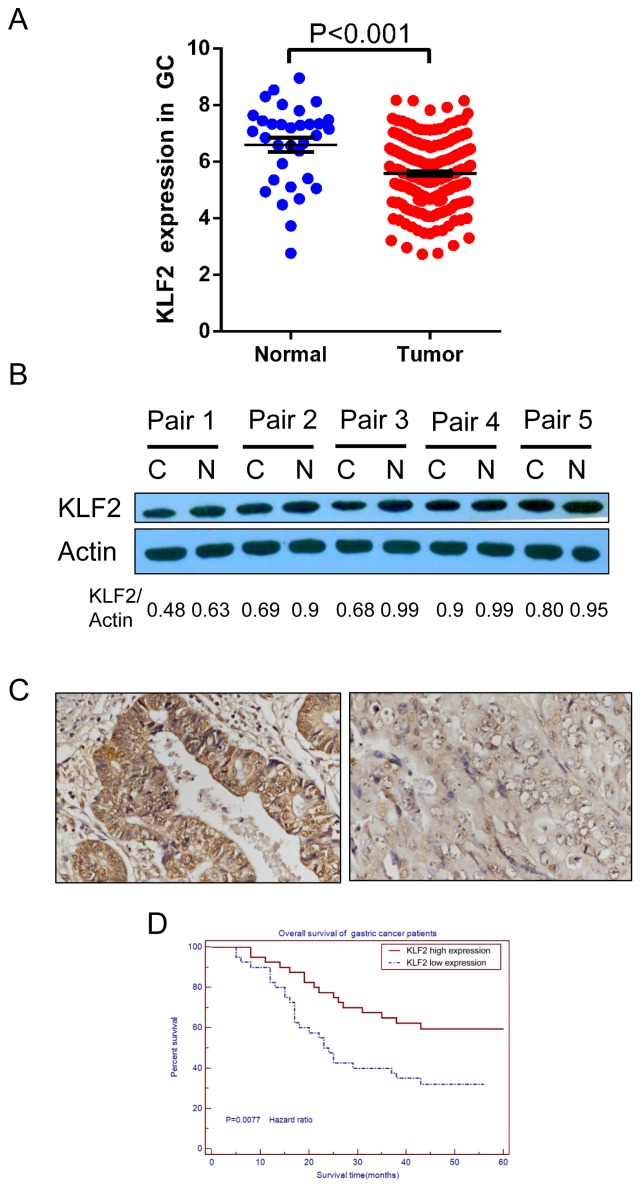
KLF2 is downregulated in human GC and its expression level is correlated with patients’ survival **(A)** KLF2 expression was examined by using The Cancer Genome Atlas (TCGA) database. Average expression level of KLF2 was lower in gastric tumor specimens (n = 249, 5.588±0.06843, SEM) compared with normal samples (n = 33, 6.598±0.2504, SEM). *** *P* < 0.001. **(B)** Western blot analysis of KLF2 protein expression level in five paired human gastric cancer samples and adjacent normal tissues. The level of KLF2 protein expression was decreased in tumor tissue when compared with that in normal tissue. C indicates cancer tissue; N indicates adjacent normal tissue. **(C)** IHC staining of KLF2 in human gastric tumor. Left, KLF2 strong expression. Right, KLF2 weak expression. **(D)** Kaplan-Meier analysis of survival in 80 patients with gastric cancers. The survival for patients with strong KLF2 expression was significantly longer than that for the patients with weak KLF2 expression. *P* = 0.0077.

To determine the expression of KLF2 in human gastric cancer, we first analyzed the gene expression profiles in GC found in The Cancer Genome Atlas (TCGA) database. We found that KLF2 expression was lower in gastric tumor samples (n = 249, 5.588±0.06843, SEM) compared with normal samples (n = 33, 6.598±0.2504, SEM) (p < 0.001, Figure 1A). We next examined the protein level of KLF2 in paired GC tissues and adjacent histologically normal tissues by using Western blot. We found that KLF2 protein expression was downregulated in gastric tumor tissues compared with the paired adjacent normal tissues (Figure 1B). Next, we systematically analyzed KLF2 expression in 80 human gastric cancer tissue samples on the tissue microarray by IHC staining. Based on the expression pattern, we divided those samples into KLF2 weak expression and strong expression groups (Figure 1C and Supplementary Table 2). We also determined the survival rate of these patients using their clinical data. Kaplan-Meier statistical analysis data of overall survival in GC patients, showed that the survival for patients with strong KLF2 expression was clearly longer, compared with the patients with weak KLF2 expression (*p* = 0.0077; Figure 1D). These results indicated that KLF2 is frequently downregulated in human GC and is closely associated with prognosis in GC patients.

### The establishment of stable KLF2-overexpressing and KLF2-knockdown GC cell lines

**Figure 2:**
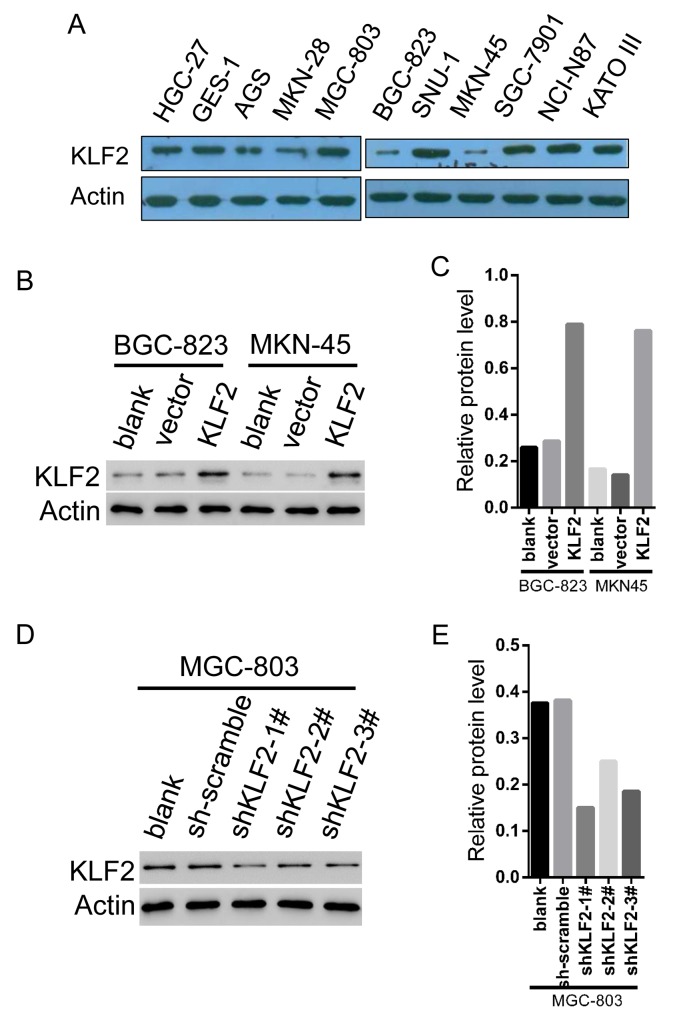
The establishment of stable KLF2 overexpressing and knockdown GC cell lines **(A)** Protein expression of *KLF2* in ten human GC cell lines and one normal gastric cell line via Western blot. MKN-45 and BGC-823 express relatively low levels KLF2 whereas MGC-803 cell line expresses relatively high levels of KLF2. **(B** and **C)** MKN-45 and BGC-823 cancer cell lines were infected with Vector and KLF2-overexpressing lentivirus. The level of KLF2 in MKN-45 and BGC-823 cell lines was verified by Western blot. **(D** and **E)** MGC-803 cell line was infected with scambled and KLF2 targeting shRNA lentivirus. Western blot to verify the level of KLF2 in the MGC-803 knockdown cell line.

To assess the biological functions of *KLF2* in GC cells, we first analyzed the mRNA and protein expression of *KLF2* in ten GC cell lines and one normal gastric cell line GES-1 via real-time PCR and Western blot (Figure 2A and Supplementary Figure 1). Among these cell lines, MKN-45 and BGC-823, which express KLF2 at relatively low levels, were selected for KLF2 overexpression via transduction with lentiviral *KLF2.* In addition, the MGC-803 cell line expresses relatively high levels of KLF2 and was chosen for KLF2 knockdown using lentivirus-mediated transduction with three different shRNAs targeted to KLF2. These infected cells then underwent puromycin selection to harvest cell lines stably expressing or deficient in KLF2. We used Western blot to verify the cellular level of KLF2 and its expression was significantly increased about four times in both MKN-45 and BGC-823 cells ectopically expressing KLF2 (Figure 2B and 2C). Likewise, infection with KLF2 shRNA lentivirus all reduced KLF2 expression in MGC-803 cells, especially shRNA 1#, compared with cells infected with the scrambled virus (Figure 2D and 2E).

### KLF2 negatively regulates cell growth

**Figure 3:**
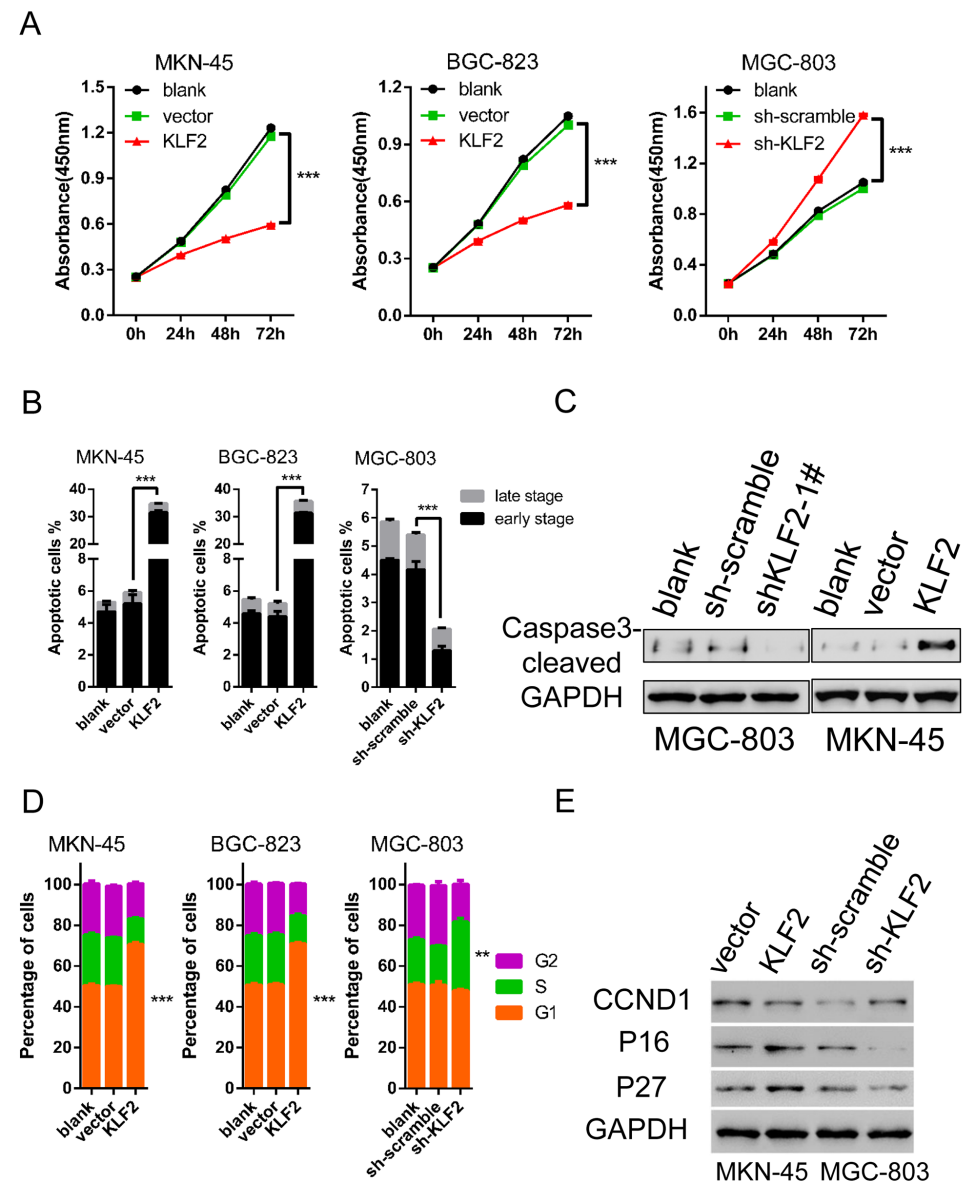
KLF2 inhibits cell proliferation and induces cell apoptosis and cell cycle arrest **(A)** Alteration of KLF2 influenced the proliferation of MKN-45, BGC-823 and MGC-803 cells by CCK8 assay at different time points. KLF2 overexpression significantly inhibited MKN-45 and BGC-823 cell proliferation. Knockdown of KLF2 enhanced cell proliferation in MGC-803 cells. *** *P* < 0.001. **(B)** Percentage of apoptotic cells, including early and late stage, in KLF2 overexpressing and deficient cell lines was determined after analyzing Annexin V and PI double labeled cells through FACS. *** *P* < 0.001. **(C)** Levels of apoptosis-associated marker protein cleaved caspase 3 was determined via Western blot in KLF2 overexpressing MKN-45 cells and KLF2 deficient MGC-803 cells. **(D)** Comparison of cell cycle distribution in KLF2 overexpressing and deficient cell lines, based on FACS analysis. **(E)** Protein expression of cell cycle regulators such as p16/ CDKN2A, p27/CDKN1B and CCND1 in KLF2 overexpressing MKN-45 cells and KLF2 deficient MGC-803 cells.

We evaluated the roles of KLF2 in cell growth kinetics by employing the cells acquired above. CCK8 assay was performed to determine of cell viability *in vitro* of these three cell lines. Results showed that ectopic expression of KLF2 significantly suppressed the proliferation of MKN-45 and BGC-823 cells (Figure 3A left and middle). Conversely, reduced KLF2 levels obviously promoted MGC-803 cell growth compared with the scrambled shRNA group (Figure 3A right). These results clearly indicated that forced expression of KLF2 led to inhibition of GC cell proliferation. To further clarify the reason for KLF2’s inhibitory impact on cell proliferation, we examined the effects of KLF2 expression on cell apoptosis and the cell cycle through fluorescence-activated cell sorting (FACS) analysis. After using FACS to analyze Annexin V and Propidium Iodide (PI) double labeling cells, we found that overexpression of KLF2 induced massive cell apoptosis (around 35% of cells) in both MKN-45 and BGC-823 cell lines (Figure 3B and Supplementary Figure 2). While in KLF2 knockdown MGC-803 cells, the percentage of cell apoptosis was lower than the control group. The FACS results were confirmed by detecting apoptosis maker proteins such as cleaved Caspase 3 and Poly ADP ribose polymerase (PARP) via Western blot (Figure 3C). In addition, FACS analysis also discovered that altered KLF2 expression influences the cell cycle distribution. In the KLF2 overexpression cells, there was about a 20% increase in the G1 phase. In contrast, upon KLF2 downregulation more cells entered S phase, roughly estimated at 15% (versus 20% in control cells, Figure 3D and Supplementary Figure 3). Therefore, KLF2 overexpression caused cell cycle arrest and its deficiency activated cells to grow faster. We used Western blot to analyze the protein expression of cell cycle regulators, and found that p16 / CDKN2A, p27 / CDKN1B were upregulated and CCND1 were downregulated in response to KLF2 overexpression (Figure 3E). Taken together, these findings demonstrated that KLF2 inhibits cell proliferation, enhances apoptosis and induces cell cycle arrest.

### KLF2 inhibits cell migration and invasion

**Figure 4:**
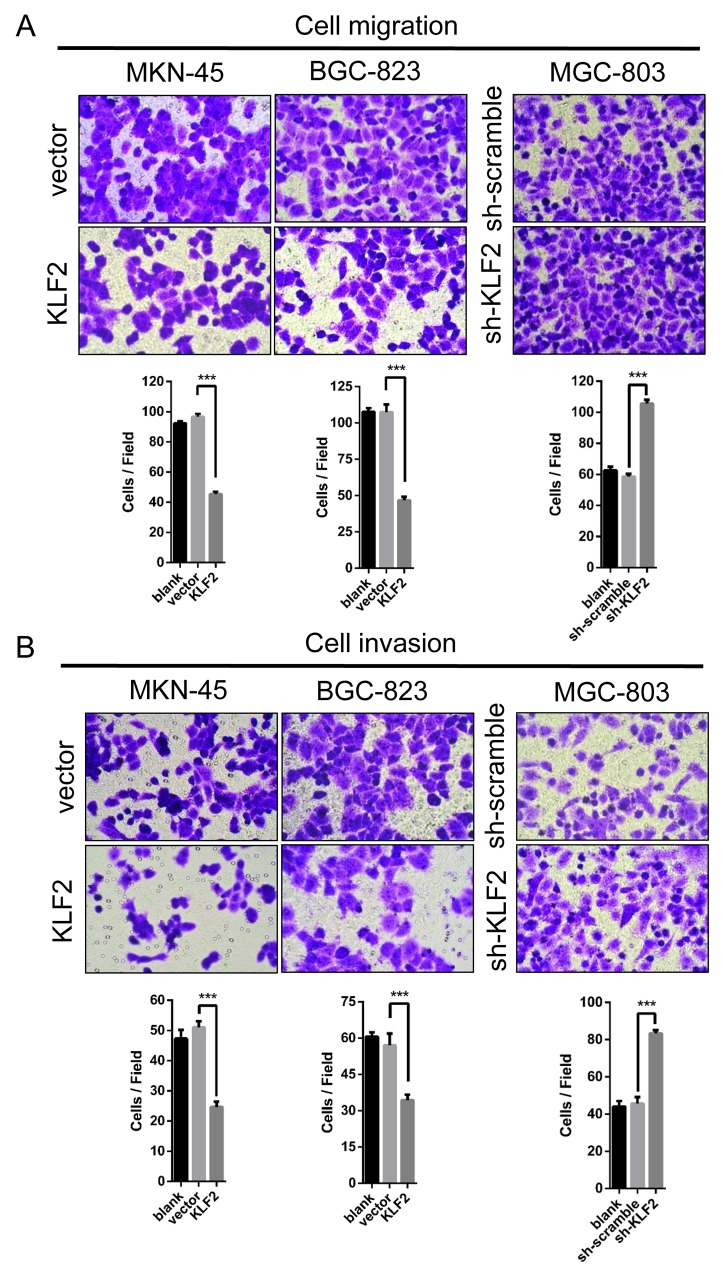
KLF2 inhibits cell migration and invasion **(A** and **B)** Transwell assay was conducted to test the effect of KLF2 on the migration and invasion of MKN-45, BGC-823 and MGC-803 cells. Number of cells were counted and shown in the panels below the corresponding pictures. Data are mean ± SD of three independent experiments. *** *P* < 0.001.

As an important hallmark of cancer, activating invasion and metastasis make cancers hard to treat and have been the main cause of patient death. Hence, we wondered whether KLF2 affects GC cell migration and invasion. We performed the transwell assay to assess GC cells’ migration and invasion with altered KLF2 expression. As shown in Figure 4A and 4B, KLF2 markedly depressed GC cell migration and invasion. These results indicated that KLF2 suppresses GC cells’ migration and invasion capability *in vitro*.

### KLF2 deficiency accelerates gastric tumorigenesis *in vitro* and *in vivo*

**Figure 5:**
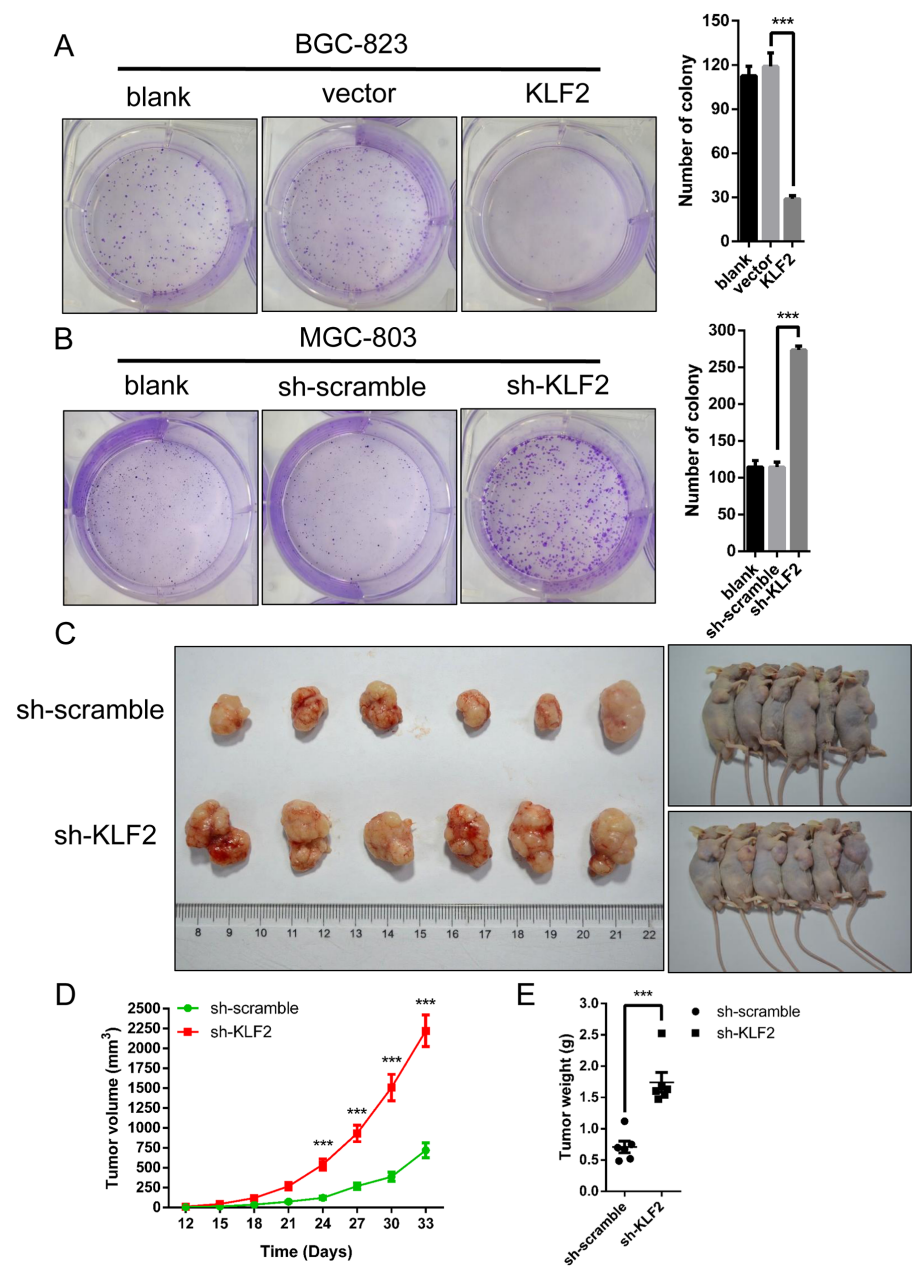
KLF2 suppresses cell growth *in vitro* and gastric tumor formation *in vivo* **(A** and **B)** Colony formation assays were performed to determine the proliferation of BGC-823 and MGC-803 cells. The colonies were counted after fixation and staining with crystal violet and are shown in the right panel. Data are mean ± SD of three independent experiments. *** *P* < 0.001. **(C)** Representative pictures showing the effects of KLF2 on tumor growth, as determined in a xenograft mouse model. KLF2 deficient MGC-803 cells were injected into nude mouse flanks. Tumor size was measured every 3 days and the growth curves were shown in **(D)**. Tumors were harvested and weighed after one month as shown in **(E)** *** *P* < 0.001.

Based on the above results, we speculated that KLF2 could suppress gastric tumorigenesis. We conducted *in vitro* colony formation assays to test our deduction. Indeed, as shown in Figure 5A, ectopic expression of KLF2 remarkably reduced the number of colonies in BGC-823 cells. Conversely, KLF2 deficiency significantly increased the number of colonies in MGC-803 cells (Figure 5B). To further testify our prediction, we used tumor transplantation mouse models to evaluate KLF2’s inhibitory effect *in vivo*. Counted cells were injected into the right hindlimb of nude mice subcutaneously and tumor size was monitored after the tumor transplantation. During the tumor development, we found that knockdown of KLF2 significantly promoted tumor formation *in vivo* (Figure 5C and 5D). The weight of these tumors also was increased in KLF2 knockdown xenografts (Figure 5E). Of note, these results were in line with the previous conclusion of CCK8 assays (Figure 3A). Collectively, impaired KLF2 expression obviously accelerated gastric tumorigenesis *in vitro* and *in vivo*.

### KLF2 induces PTEN expression and inhibits AKT-mTOR activity

**Figure 6:**
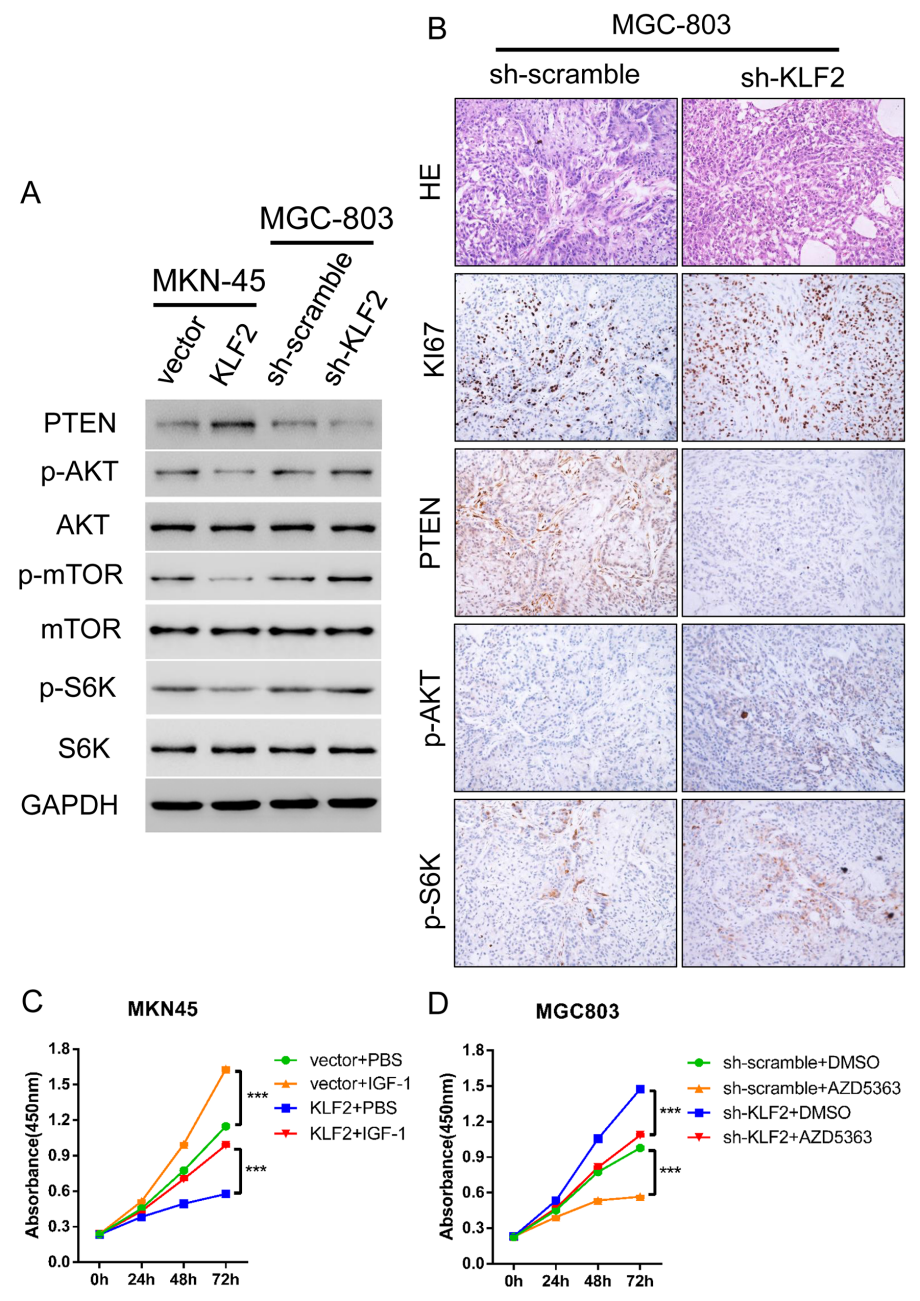
KLF2 activation of PTEN results in reduced Akt-mTOR signaling in GC cells **(A)** KLF2 overexpressing MKN-45 cells and KLF2 deficient MGC-803 cells were subjected to Western blot to determine the expression of PTEN, and p-AKT signaling and its downstream substrates p-mTOR and p-S6K. **(B)** HE staining and immunohistochemical staining of KI67, PTEN, p-AKT and p-S6K in KLF deficient MGC-803 cell-derived xenograft tumors. **(C** and **D)** Administration of AKT pathway activator IGF-1 and AKT inhibitor AZD5363 significantly reversed the cell proliferation phenotype determined by the CCK8 assay. *** *P* < 0.001.

To uncover the potential mechanism for KLF2’s negative regulation of GC, we searched for the main signaling pathways disturbed by KLF2 alteration in GC. GSEA was performed on the TCGA GC dataset [15] to identify the relevant dysregulated molecular pathways. We discovered that the expression of *KLF2* genes was negatively associated with cell cycle, cell apoptosis and PTEN/AKT pathways (Supplementary Figure 4A-4C). It is well known that PTEN is a classic tumor suppressor and its aberrant expression has often been found in various cancers. We used Q-PCR and Western blot to validate whether PTEN and its downstream targets were altered in response to KLF2 expression level. As our results show, the mRNA and protein levels of PTEN were in concert with KLF2 expression (Figure 6A and Supplementary Figure 5). Likewise, PTEN was decreased in KLF2 deficient xenograft tumors by IHC staining (Figure 6A). Furthermore, we found that levels of phosphorylated AKT, as well as phosphorylated mTOR and S6K, inversely correlated with KLF2 and PTEN (Figure 6A), suggesting that KLF2 expression level may regulate PTEN/AKT pathway activity. Immunohistochemical staining indicated that p-AKT and p-S6K were increased in KLF2 deficient xenograft tumors (Figure 6B). In addition, KI67 staining also confirmed previous conclusion that knockdown KLF2 improved cell proliferation (Figures 3A and 6B). Based on these findings, we further wondered whether KLF2’s effect could be reversed by administration of IGF-1, which results in activation of AKT, or AKT inhibitor AZD5363. As expected, we found that cell viability is rescued when we added IGF-1 to the culture medium (Figure 6C). Likewise, cell viability was significantly suppressed in the AZD5363 containing medium (Figure 6D). Collectively, these results strongly demonstrated that KLF2 suppresses gastric tumorigenesis probably through inhibition of AKT-mTOR signaling.

## DISCUSSION

In this study, we thoroughly explored the expression and potential effect of KLF2 in human gastric tumorigenesis. We first found different expression patterns between normal and neoplastic gastric tissues. To be precise, KLF2 was highly expressed in the cytoplasm and nucleus of epithelial cells in normal tissue, implying KLF2’s potential function in maintaining homeostasis of normal epithelium. By contrast, we discovered that KLF2’s expression level was substantially lower in both gastric cancer cell lines and human tumor specimens. Moreover, our clinical analysis revealed negative correlation between KLF2 level and patient survival. Furthermore, ectopic expression of KLF2 largely suppressed GC cell growth, migration and invasion *in vitro* and tumorigenesis. Mechanistic analysis found that KLF2 induced PTEN expression at both the mRNA and protein level. KLF2 also significantly suppressed AKT-mTOR signaling which is downstream of PTEN. Therefore, we provided the first evidence and potential mechanism that KLF2 expression level plays an important role in gastric tumorigenesis and that KLF2 associated signaling could be a therapeutic target in GC.

The mammalian gastrointestinal tract is a special system which has the most rapid self-renewal rate throughout the entire life span. Proper stem cell proliferation and differentiation facilitate the formation of the stringent cell hierarchy which is tightly regulated by complex signaling pathways [16, 17]. Once the delicate balance is broken, it will lead to diseases like cancer. Previous research revealed several main pathways critical in controlling gastric homeostasis, such as the nuclear factor-κB (NF-κB), EGFR/MAPK, Wnt/β-catenin, Hedgehog, Notch and bone morphogenetic protein (BMP) signaling pathways [18]. However, the detailed mechanism of how these signaling cascades operate to regulate gastrointestinal epithelial cell proliferation and differentiation still needs to be understood.

Currently, there is little known about KLF2’s expression and its potential contribution to gastric cancer formation. Previous studies have shown KLF2 expression is downregulated in many human cancers, such as ovarian [9], prostate [10] and lung [12] cancer. Functionally, KLF2 acted as a tumor suppressor which inhibits cell proliferation and induces cell apoptosis. Unexpectedly, KLF2 was found to be upregulated in HCC [14] and appeared to be an oncogene. In the present research, we observed that KLF2 was remarkably reduced in gastric tumors, clinical and laboratory findings clearly showed that KLF2 acts as a tumor suppressor in human GC. These results suggested that KLF2 play complicated roles in cancer development. We think it could be attributed to the specific context and diverse binding partners of KLF2 in different tissue resulted in distinct downstream signaling. Because KLF2 has not been found mutational inactivation in tumors, it is not hard to raise the question of what mechanism regulates KLF2 expression in human GC. Previous research has shown that one of the Polycomb group proteins, the histone methyltransferase EZH2, represses KLF2 transcription directly through epigenetic silencing [13]. Indeed, EZH2 has similar effects on tumor cells as KLF2, such as inducing apoptosis and restraining the cell cycle. It is well worth exploring the roles of EZH2 and related epigenetic changes in human GC. In addition, another Polycomb group protein, SUZ12, has been reported to regulate KLF2’s expression [19]. Recently, several studies have found that some long noncoding RNAs like ZFAS1 [20], TINCR [21] and LINC00673 [22] inhibit KLF2 expression through transcriptional regulation. All of these findings support our result that KLF2 functions as a tumor suppressor.

Although the specific mechanism by which KLF2 regulates tumorigenesis is unclear, there is evidence suggesting that KLF2 influences the cell cycle [12, 20]. In our study, we also discovered that ectopic expression of KLF2 obviously induced cell cycle arrest and cell apoptosis which was mediated by p16/CDKN2A and p27/CDKN1B. In addition, KLF2 expression in GC was negatively correlated with cell cycle proteins, apoptotic mediators, and the PTEN/AKT pathway. It is well known that PTEN suppresses AKT-mTOR signaling which plays critical roles in tumor development and progression [23, 24]. PTEN expression has been reported diminished in human gastric cancer [25]. Our data showed that KLF2 induced PTEN expression and AKT-mTOR signaling was significantly upregulated in KLF2-deficient cells and tumors. Moreover, activation or suppression of this signaling was very similar with the effect of KLF2 knockdown or overexpression, suggesting that PTEN- AKT- mTOR acts as a main downstream signaling mediator of KLF2. Of note, it will be interesting to investigate how KLF2 regulates PTEN expression and whether, as a transcription factor, KLF2 directly enhances PTEN expression.

In conclusion, we found that expression of KLF2 was relatively high in normal gastric epithelium, and was often downregulated in gastric cancer cell lines and tissues. Impaired KLF2 expression led to tumor growth promotion *in vitro* and *in vivo* by activating AKT-mTOR signaling through downregulating PTEN. Our results support the possibility that KLF2 acts as a tumor suppressor in gastric tumorigenesis and serves as a potential therapeutic target.

## MATERIALS AND METHODS

### Clinical tissue samples

Tissue microarray of eighty cases of human gastric cancer who are aged from 22 to 80 years (average 55 years) was purchased from Shanghai Outdo Biotech Co. Ltd (Shanghai, China), which contains 77 adenocarcinoma, 3 mucinous adenocarcinoma. Fifteen gastric cancer tissue samples and paired paracancerous tissue samples (confirmed by pathology) were all from patients after surgery in Shanghai East Hospital Affiliated to Tongji University, and informed consent was obtained from all these patients. This study protocol was approved by the Institutional Review Board of Shanghai East Hospital. Gastric tissue microarray were used for immunohistochemical (IHC) assay (described below) and corresponding clinicopathologic variables were collected for further analysis. The KLF2 expression was graded as follows: 0 (<5%), 1 (5%-24%), 2 (25%-49%), 3 (50%-74%), 4 (>75%). A score of 0-2 was taken as weak KLF2 expression, and score 3-4 as strong.

### Cell lines and transfection

Gastric cancer cell lines: HGC-27, SNU-1, SGC-7901, NCI-N87, KATOIII, AGS, MKN-28, MKN-45, BGC-823, MGC-803 and GES-1 were purchased from the Chinese Academy of Sciences (Shanghai, China), and were cultured in corresponding medium containing 10% fetal bovine serum (FBS). All the cell lines were kept at 37°C in 5% CO_2_. To obtain KLF2 over-expressing cells, BGC823 and MKN45 cells were infected with lentivirus encoding GFP-KLF2 (KLF2) or GFP (as control) (Shanghai Genechem Co., LTD.). MGC803 cells were infected with lentivirus encoding shRNA KLF2 or scrambled shRNA used as negative Control Duplex (Hanbio Biotechnology Co., Ltd.) to obtain KLF2 knockdown cells. Three different shRNA sequences were used: shRNA1: sense5’-GATCCGCGGCACCGACGACGACCTCAATTCAAGAGATTGAGGTCGTCGTCGGTGCCGTTTTTTC-3’, antisense 5’- AATTGAAAAAACGGCACCGACGACGACCTCAATCTCTTGAATTGAGGTCGTCGTCGGTGCCGCG-3’; shRNA2: sense5’- GATCCGCCTACACCAAGAGTTCGCATCTGAATTCAAGAGATTCAGATGCGAACTCTTGGTGTAGGTTTTTTC-3’, antisense 5’- AATTGAAAAAACCTACACCAAGAGTTCGCATCTGAATCTCTTGAATTCAGATGCGAACTCTTGGTGTAGGCG-3’; shRNA3: 5’- GATCCGCGCTGCACATGAAACGGCACATGTATTCAAGAGATACATGTGCCGTTTCATGTGCAGCGTTTTTTC-3’, antisense 5’- AATTGAAAAAACGCTGCACATGAAACGGCACATGTATCTCTTGAATACATGTGCCGTTTCATGTGCAGCGCG-3’. The transfected cells were selected in culture medium plus puromycin (1.0 μg/ml) for 3 weeks to obtain stable KLF2-overexpressing and KLF2-deficient cells.

### Real-time PCR

Total RNA from specimens and gastric cancer cell lines was extracted using TRIzol Reagent (Invitrogen). RNA was reverse transcribed into cDNA using a RT-PCR kit (Fermentas). cDNA was used for real-time PCR assay performed with SYBR Green PCR kit (Thermo) carried out on an ABI-7300 (Applied Biosystem) thermal cycler. GAPDH was used as internal control. The results were analyzed by ABI Prism 7300 SDS Software and expressed as threshold cycle (CT) values. The specific primer sequences for real-time PCR are listed in Supplementary Table 1.

### Western blotting and antibodies

BGC-823, MGC-803, MKN-45 cells and mouse tumor tissues were lysed with RIPA buffer (Solarbio, Shanghai, China) supplemented with Protease Inhibitor Cocktail and Protein Phosphatase Inhibitor (Solarbio, Shanghai, China). The concentration of protein samples was determined by using the BCA Protein Quantitation Kit (Promega, USA). Samples were separated by SDS-PAGE; proteins were then transferred onto Nitrocellulose membrane filters (Millipore, USA) by wet blotting. After treatment with skim milk, membranes were incubated with primary antibody overnight at 4°C and then with corresponding secondary antibody for 1 h at room temperature. The Odyssey system (LI-COR, USA) was used to measure the protein signal.

Anti-KLF2 (1:1000, ab194468), anti-Caspase3 (1:1000, ab44976), anti-CCND1 (1:1000, ab134175), anti-p27 (1:1000, ab32034), anti-p16 (1:1000, ab201980), anti-active Caspase-3 (1:1000, ab2302), anti-Cleaved PARP (1:1000, ab32064), anti-PARP (1:1000, ab32138), anti-S6K (1:1000, ab32529), and anti-p-S6K (1:1000, ab126818) were purchased from Abcam (Cambridge, MA, USA), and anti-GAPDH (1:3000, #5174), anti-p-AKT (1:1000, #9271), anti-AKT (1:1000, #9272), anti-PTEN (1:1000, #9552), anti-mTOR (1:1000, (#2972) and anti-p-mTOR (1:1000, #5536) were purchased from Cell Signaling Technology (Danvers, MA, USA).

### Cell proliferation assays

Cell proliferation was detected by the CCK-8 assay. Briefly, 3 x103 cells per well were seeded in 96-well plates in triplicates. 100 μl of the 1:10 Cell Counting Kit-8 dilution (CCK-8) (SAB CP002) was added after transient transfection with lentivirus for 0, 24, 48 and 72 h, and incubated at 37°C for 1 h. The absorbance at a wave-length of 450nm was detected by microplate reader.

### Colony formation assay

Colony formation assay was performed to determine the effect of KLF2 on the cloning capability of gastric cancer cells. BGC-823, MGC-803 and MKN-45 cells stably transfected with lentivirus were cultured 500 cells / well in 6-well plates for two weeks. Visible cell colonies were counted after staining with crystal violet solution.

### Cell apoptosis assays

BGC823, MGC803 and MKN45 cell apoptosis was assessed by dual staining with FITC-Annexin V (eBioscience 88-8007) and Propidium iodide (PI) after transfection with lentivirus for 48h according to the manufacturer’s instructions and cells were analyzed with a flow cytometry (BD, Accuri C6).

### Cell cycle analysis

The cells were collected and washed and then fixed in 70% ethanol. After incubation with RNase (Solarbio, R8020-25;1mg/ml) for 30 min at 37°C, the cells were stained with Propidium iodide (PI, 50μg/mL) in the dark for 10 min. Finally, the cell cycle phase was detected by flow cytometry (BD, Accuri C6).

### Transwell assays

Cell invasion and migration were examined using the transwell assay. 0.3 ml of 2 × 10^5^ Cells/ml was suspended in serum-free medium in the top of transwell chambers (Coming, 356234; for invasion assay, the chamber was coated with 80μl Matrigel 30 min before assay in), 0.5 ml medium containing 10% FBS was added to the lower chambers. After cultured for 12 h at 37°C, cells on the upper surface were removed using a swab and cells on the lower surface of the membrane were fixed in 4% paraformaldehyde for 10 minutes, then dyed with 0.5% crystal violet for counting.

### Tumor xenograft model

12 male BALB/c nude mice (Age 4-6 weeks, obtained from the animal center of East China Normal University (Shanghai, China)) were randomly divided into two groups, and were subcutaneously injected with 4×10^6^ MGC-803 cells stably transfected with shRNA#1-KLF2 or scrambled shRNA. Tumor growth was examined for 33 days and recorded. Tumor volumes were measured every 3 days (volumes were calculated by the equation: V = 4/3 × 3.14 × (d/2)^3^ (V, volume; d, diameter). At the end of the experiment, the mice were euthanized and photographed. Tumors were excised, weighed, and divided into several sections, some for KLF2 detection by PCR and Western blot, while others were fixed in 4% paraformaldehyde for HE and IHC staining. All the experiments were approved by the Ethics Committee of Animal Experiments of East China Normal University in Shanghai.

### Histology

Tumors removed from the mice were fixed in 4% paraformaldehyde overnight, embedded in paraffin, sectioned at 4 μm thickness and then were stained with HE and immunohistochemistry (IHC) assay. For IHC, sections were incubated with the primary antibody at 4°C overnight and the secondary antibody at room temperature for 30 min before staining. Slides were observed using a microscope (OLYMPUS, CX41) and staining was expressed by positive area. The primary antibodies used in the assay are listed as follows: anti-Ki67 (1:100, Thermo Fisher Scientific, RM-9106-S1), anti-PTEN (1:100, #9559), anti-p-AKT (1:100, #4060), anti-p-S6K (1:100, #9206) were purchased from Cell Signaling Technology (Danvers, MA, USA).

### Statistical analysis

Statistical analysis was conducted by SPSS 20.0 software and p < 0.05 was considered statistically significant. Statistical significance of difference between groups was performed by Student’s t test or ANOVA. Kaplan–Meier survival analysis and log-rank statistics were performed to compare survival curves.

## SUPPLEMENTARY MATERIALS FIGURES AND TABLES



## Abbreviations

KLFs: Krüppel-like factors
IGF-1: insulin-like growth factor-1
GC: gastric cancer
NSCLC: non-small-cell lung cancer
HCC: hepatocellular carcinoma
FACS: fluorescence-activated cell sorting
PI: propidium iodide
PARP: poly ADP ribose polymerase
NF-kB: nuclear factor-Kappa B
BMP: bone morphogenetic protein
GSEA: Gene Set Enrichment Analysis
