# Supplementary material for: Krüppel-like factor 2 suppresses human gastric tumorigenesis through inhibiting PTEN/AKT signaling
Source: Oncotarget. 2017 Nov 1;8(59):100358–70. doi: 10.18632/oncotarget.22229 (PMC5725026; doi:10.18632/oncotarget.22229)
Supplement: Supplementary file 1 [file oncotarget-08-100358-s001.pdf]

Krüppel-like factor 2 suppresses human gastric tumorigenesis through inhibiting PTEN/AKT signaling

SUPPLEMENTARY MATERIALS

REFERENCES

1. Subramanian A, Tamayo P, Mootha VK, Mukherjee S, Ebert BL, Gillette MA, Paulovich A, Pomeroy SL, Golub TR, Lander ES, Mesirov JP. Gene set enrichment analysis: a knowledge-based approach for interpreting genome-wide expression profiles. Proc Natl Acad Sci U S A. 2005; 102:15545-15550.

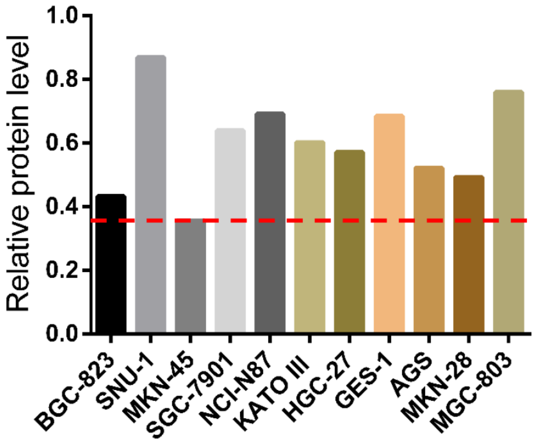

Supplementary Figure 1: The mRNA level of KLF2 in normal cell line (GES-1) and gastric cancer cell lines (the other ten cell lines). The KLF2 expression level is relative to GAPDH. Among them, KLF2's expression in MKN-45 and BGC-823 cell lines are lower while in MGC-803 and SNU-1 are higher.

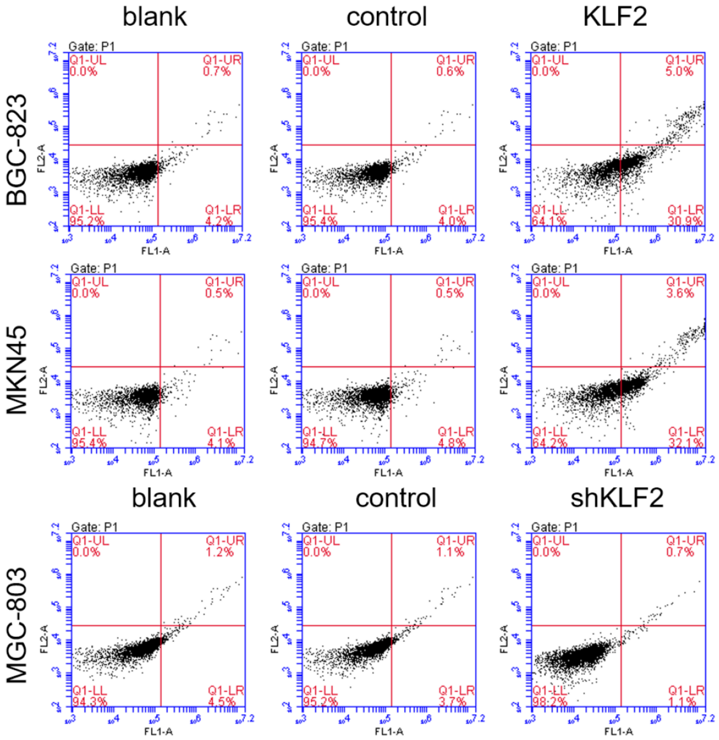

Supplementary Figure 2: Representative FACS results of three independent experiments show that KLF2 expression suppressed cell apoptosis.

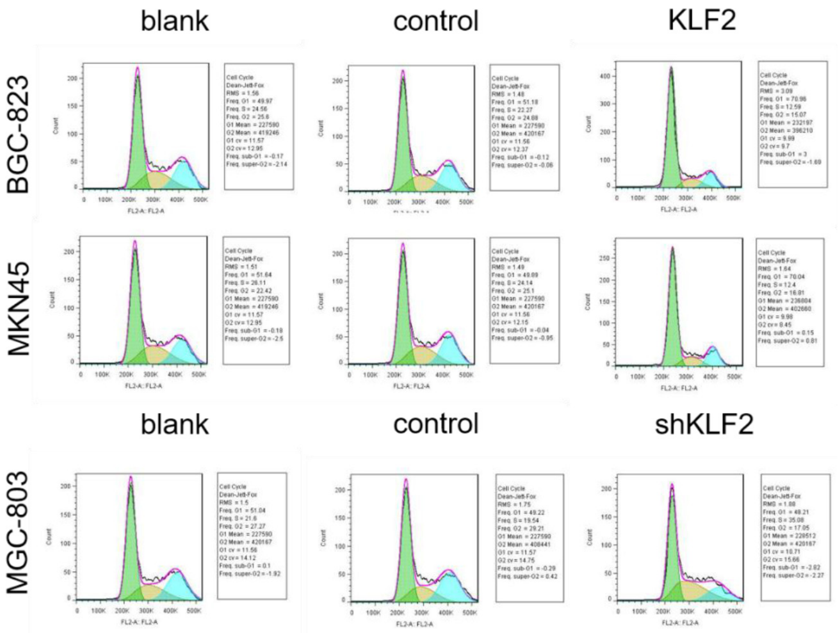

Supplementary Figure 3: Representative FACS results of three independent experiments show that KLF2 expression inhibited cell cycle progression.

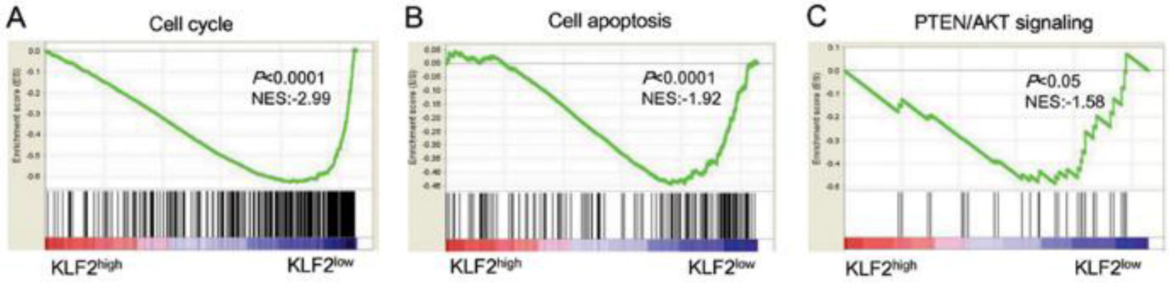

Supplementary Figure 4: GSEA (Gene Set Enrichment Analysis) was performed to identify pathways and biological process that are enriched in genes differentially expressed as previously described [1]. GSEA analysis in GC patients with higher KLF2 expression versus lower KLF2 expression based on TCGA GC dataset. NES, normalized enrichment score. Cell cycle **(A)**, cell apoptosis **(B)** and PTEN/AKT signaling pathways **(C)** have negative association with KLF2-higher expression.

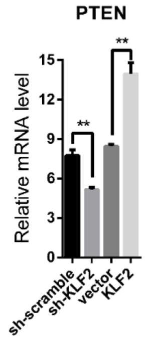

Supplementary Figure 5: Q-CPR was performed to determine PTEN expression level in KLF2 overexpressing MKN-45 cells and KLF2 deficient MGC-803 cells.

Supplementary Table 1: Primer sequences for Real-Time PCR

Gene	Product size (bp)	Primer sequence (forward)	Primer sequence (reverse)
KLF2	186	CTGCTCTGTCTGCCTCCAAG	CTGCTCTCCAGGTGGGTTTC
PTEN	141	TCAGGCGAGGGAGATGAGAG	CGAAGAGGAGGCGAGAAACG
P53	135	CCACCATCCACTACAACTAC	AAACACGCACCTCAAAGC
P21	249	TAGCAGCGGAACAAGGAG	AAACGGGAACCAGGACAC
P27	220	GCTTGCCCGAGTTCTACTAC	GCAGGTCGCTTCCTTATTCC
P16	105	GGGTTTTCGTGGTTCACATCC	CTAGACGCTGGCTCCTCAGTA
P15	209	TGTCTGCTGAGGAGTTATGG	TCTGCTATCTGGTGGAGTTG
CCNB1	111	AATAAGGCGAAGATCAACATGGC	TTTGTTACCAATGTCCCCAAGAG
CCND1	135	GCTGCGAAGTGGAAACCATC	CCTCCTTCTGCACACATTTGAA
GAPDH	110	CACCCACTCCTCCACCTTTG	CCACCACCCTGTTGCTGTAG

Supplementary Table 2: The correlation between KLF2 expression and clinicopathological factors in 80 gastric cancer patients

Characteristics	Total (n=80)	KLF2 expression		*P* value
		Strong (%), num. of cases	Weak (%), num. of cases	
Age (years)				
≥55	40	21 (43.8)	19 (59.4)	0.171
<55	40	27 (55.2)	13 (40.6)	
Gender				
Male	64	36 (75)	28(87.5)	0.171
Female	16	12 (25)	4 (12.5)	
Histological subtype				
Adenocarcinoma	77	46 (95.8)	31 (96.9)	0.810
Mucinous adenocarcinoma	3	2 (4.2)	1 (3.1)	
Grade				
1,2	36	14 (29.2)	22 (66.8)	0.000*
3,4	44	34 (70.8)	10 (31.2)	
TNM stage				
I + II	57	31 (64.6)	26 (81.2)	0.107
III + III	23	17 (35.4)	6 (18.8)	
